# Habit

**Published:** 1875-10

**Authors:** 


### HABIT.

"I trust everything," said Lord Brougham, "to habit, upon which, in all ages, the lawgiver as well as the schoolmaster, has mainly placed reliance; habit, which makes everything easy, and casts all difficulties upon the deviation from a wonted course. Make sobriety a habit, intemperance will be hateful; prudence a habit, and reckless profligacy will be as contrary to the nature of a child, grown or adult, as the most atrocious crimes are to any of your lordships. Give a child the habit of sacredly regarding the truth; of carefully respecting the property of others; of scrupulously abstaining from all acts of improvidence which can involve him in distress, and he will just as likely think of rushing into an element in which he can not breathe, as of lying, cheating, or swearing."

---

The Mother.—Despise not your mother when she is old. Age may wear and waste a mother's beauty, strength, senses, and estate; but her relation as mother is as the sun when it goes forth in its might, for it is always in the meridian, and knoweth no evening. The person may be gray-haired, but the motherly relation is ever in its bloom. It may be Autumn, yea, Winter; but with the mother, as mother, it is always Spring. Alas! how little do we appreciate a mother's tenderness while living! How heedless are we in youth of all her anxieties and kindness! But when she is dead and gone—when the cares and the coldness of the world come withering to our hearts—then it is that we think of the mother we have lost.

Girls at Play.—There is hardly another sight in the world so pretty as that of a company of young girls—almost women grown—at play, and so giving themselves up to their airy impulse that their tip-toes barely touch the ground. Girls are so incomparably wilder and more effervescent than boys, more untamable, and regardless of rule and limit, with an ever-shifting variety, breaking continually into new modes of fun, yet with a harmonious propriety through all. Their steps, their voices, appear free as the wind, but keep consonance with a strain of music inaudible to us. Young men and boys, on the other hand, play, according to recognized law, old traditionary games, permitting no caprices of fancy, but with scope enough for the outbreak of savage instincts; for, young or old, in play or in earnest, man is prone to be a brute.

